# Deductibles in Health Insurance, Beneficial or Detrimental: A Review Article

**Published:** 2020-05

**Authors:** Iman MIRIAN, Mohammad Javad KABIR, Omid BARATI, Khosro KESHAVARZ, Peivand BASTANI

**Affiliations:** 1. Student Research Committee, School of Management and Medical Informatics, Shiraz University of Medical Sciences, Shiraz, Iran; 2. Health Management and Social Development Research Center, Faculty of Medicine, Golestan University of Medical Sciences, Gorgan, Iran; 3. Education and Development Center, Hospital Research Center, Iran University of Medical Sciences, Tehran, Iran; 4. Health Human Resources Research Center, School of Management and Medical Informatics, Shiraz University of Medical Sciences, Shiraz, Iran

**Keywords:** Deductibles, Insurance, Health

## Abstract

**Background:**

One of the ways for cost-sharing in health system that has been taken into consideration in recent years in some developed countries is paying deductibles. In case of using deductibles, the insured people more carefully and accurately will use health care services, and potentially many unnecessary costs will be avoided.

**Methods:**

To investigate the evidence of deductibles in health systems across the world, a literature review was conducted by searching the materials published in databases including ISI web of science, PubMed, Scopus and also Google scholar search engine from 2000 to 2017. Besides the related websites including WHO and the World Bank were searched. Inclusion criteria were studies carried out only in health insurance, English language, and the year of the study.

**Results:**

The most important positive impacts of deductibles were decrease in utilization of different services, high profitability for the young and healthy people, lower health benefit claims by the insured people, and increase in financial profitability of health insurance organization. Besides, the most negative impacts increase in out of pocket burdens and also higher hospitalization over time.

**Conclusion:**

Deductible plans have their own advantages and disadvantages for the insured and insurance organizations in terms of financial dimensions as well as utilization of health services, and explicitly none of these plans can be flawless. Given the increasing costs of health systems and the potential moral hazard of insured persons, it seems these systems sooner or later should inevitably move towards new cost-sharing plans, including deductibles.

## Introduction

Today, due to advancement of technology in various fields of manufacturing and services, the growing costs have become inevitable. This issue is more eminent in health sector due to the application of diverse diagnostic, therapeutic and rehabilitation methods, as well as using advanced, expensive and complicated equipment for a significant proportion of the population. In facing increasing costs of health care systems around the world, limiting the costs is the common goal of most reforms conducted in health care systems. Meanwhile, one of the major issues that health care systems, and in particular the insurance organizations, have faced over the past decade, is the moral hazard issue. Moral hazard means changes in health-related behaviors and the amount of health care services consumed due to having health insurance (1). Moral hazard has negative impacts on society and on the health care system. The most important of these impacts are reducing the welfare of people - especially the poor- due to non-optimal allocation of resources, increased premiums and reduced insurance coverage, negative external effects, reduced technical efficiency and allocative efficiency, as well as a reduction in using the benefits of risk pooling (2).

In response to this issue, insurance organizations have adopted different strategies to control unnecessary patients’ demands and increase resources for health care financing, most notably cost-sharing when receiving health care services in the forms of coinsurance, copayment, deductible, payment cap, and no-claims bonus (3).

Among the above methods, one of the ways that have been taken into consideration in recent years in some of the world developed countries is paying deductibles. In this method, the insured person must pay a certain and fixed amount for covered health care services before the insurance organization starts to pay (4). The philosophy of deductibles is that most insured persons can afford low expenses of visits, medications, etc. without suffering much pressure. The reason for using this kind of cost-sharing was that insurers, physicians, and many other people believed that if insurers involve in paying for these costs from the very beginning of health costs, this would increase excessive use of medical services and consequently increases health care costs (5).

In case of using deductibles, since the insured people are obliged to participate fully in paying their costs until they reach the deductibles amounts, more carefully and accurately they will use health care services, and potentially many unnecessary costs will be avoided. Besides, since insurance organizations spend a large part of their expenses on calculation, maintenance, and reimbursement of small amounts claimed by insured persons, this method can reduce many of these additional administrative costs. On the other hand, in many cases, availability of new medical equipment and procedures is essential for treatment of people, but insurers are not able to provide them due to lack of resources, and therefore there will be a shortage of resources for more severe illnesses; a challenge that many health insurance organizations are currently facing. Indeed, by not paying for unnecessary services, insurers will be able to redistribute these resources to more cost-effective and necessary services by optimally allocating resources to other priorities of the organization. Therefore, determining appropriate deductibles can be a great success for the patient, the insurer and in general the health care system.

The experience of using deductibles and other forms of cost-sharing date back to the late 1940s in the United States. The four countries of the United States, the Netherlands, Switzerland, and Germany are pioneers in using this method. In the United States, there are two main types of deductibles in health care system. The first type which is the traditional and common model of deductible is provided by health maintenance organizations, preferred provider organizations and point of service plans, both individually and in family (6). The second type of deductible is high deductible health plans. These plans enjoy more deductible amounts and more discounts in premium (7).

Since Jan 2008, a compulsory deductible plan has been applied in the Netherlands. In addition, insured persons can opt for a voluntary deductible plan besides a compulsory deductible plan. To compensate such an increase in financial risk, the insured persons benefit from premium discounts (8).

In Switzerland, insured people are faced with an annual compulsory deductible and are also required to pay 10% of the charges surplus to their deductibles as copayment. There are, of course, other options, such as to choose a more amount for deductibles in return for more reduction in the premium for insured persons (9).

In Germany, the deductibles were provided by health social insurance companies since 2007 and in the form of optional tariffs (10).

Considering the above, this study aimed to determine the advantages and disadvantages of implementing deductible plans based on the experiences of the pioneering countries.

## Materials and Methods

To investigate the evidence of deductibles in health systems across the world, a literature review was conducted by searching the materials published in databases including ISI web of science, PubMed, Scopus from 2000 to 2017. In addition, to ensure that all the studies considered for the study are covered, the Google Scholar search engine was used. Inclusion criteria were studies carried out only in health insurance, English language, and the year of the study.

To perform the literature review in the first phase, using the keywords, the search strategy was formulated as described in Table 1 and then it was used to search the above-mentioned databases and the related websites including WHO and the World Bank to extract related studies published in this area.

**Table 1:** Search Strategy and key words used in this study

***Search Strategy***	
Search Engines and Databases: Google Scholar, PubMed, ISI web of science, Scopus	
Limits: Language (only resources with at least an abstract in English and full-text in English or Persian)	
Date: From 2000, Jan, 1 up to 2017, Dec, 30	
Strategy: #1 AND #2	
“health insurance “OR “health insurer “OR “health insurance organization”	#1
deductible OR moral hazard OR cost-sharing OR premium	#2
impact OR result OR conclusion	#3

At this stage, to access the most relevant articles in terms of the purpose of the research, using the endnote software for resource management, first using the keywords inserted in the search strategy, the title of the related papers were registered and, in the next step, the titles were refined and the most relevant papers were selected.

Then, as the articles might have been repeatedly selected, duplicate titles were removed after reviewing the titles. Next, the abstracts of the selected papers were studied and those which appeared to be in line with the purpose of the research were inserted into the software. Finally, the full texts of the articles were studied and all the information was extracted using the data collection form. All data collection forms, including the paper’s title, authors’ name, publication date, the time and place of the research, the purpose of the research, methodology, the findings and conclusions were analyzed and the outputs were tabulated.

## Results

Overall, 377 articles were found after searching the databases using the keywords. In the next step, repeated and unrelated studies (154) were excluded after the initial screening (title review), and 183 studies were entered into the secondary screening (abstract review) process. At this stage, two researchers separately reviewed the abstracts of the articles.

**Fig. 1:**
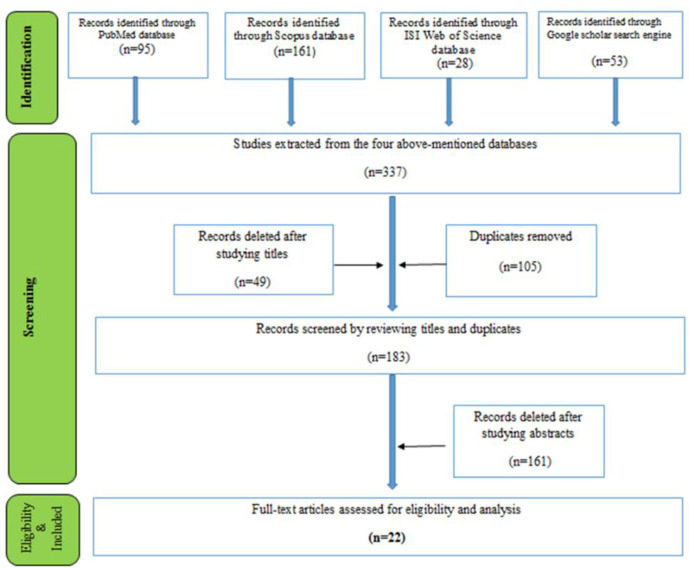
PRISMA flow diagram

To improve the accuracy and quality of the study, the results of the evaluation at this stage were reviewed by two researchers in a meeting; a third assessment was carried out to resolve the differences. The numbers of 161 papers were excluded after studying the abstracts and finally, 22 full-texts were included for reviewing the contents (Fig. 1).

From 22 selected papers that their full texts were studied, 11 studies were about the impacts of deductibles on the changes in insured persons’ behavior and the utilization of their services, 7 studies were about financial effects of deductibles, and 4 studies were about the financial effects of deductibles as well as the influence of deductibles on the insured persons’ behavior and the utilization of the service.

Of these, 13 studies were related to the United States, five studies were related to the Netherlands, three studies were related to Germany, and one study was related to Canada. Table 2 shows the main findings of these studies in the form of three general impacts of deductibles on the utilization of the insured, the financial impacts on the insured persons, and the financial impacts on insurance organizations. In addition, the most positive and negative impacts of deductibles on the three mentioned groups are shown in Table 3.

**Table 2:** Deductible plan impacts and their subcategories

***Deductible plan impacts***	***Subcategories of deductible plan impacts***	***Country***	***Reference***
Impacts on utilization of the insured	-A small effect of a moderate income-based deductible on overall access to medicines and utilization of other health services	Canada	(11)
	-The positive effect of high-deductible health plans on lowering of health care utilization	The USA	(12)
	-The highest increase in noncompliance with specialty referrals among children and patients with chronic conditions	The Netherland	(13)
	-Reduction in diagnostic imaging among high deductible health plan enrollees	The USA	(14)
	-Decrease in the probability of visiting a general Practitioner	Germany	(10)
	-Being ineffective on the probability of visiting a specialist		
	-Fair relative declines in total office visits and visits for higher-priority and lower-priority chronic illnesses.		
	- No significant differences in the rates of visits for acute higher priority or lower-priority conditions or preventive laboratory tests	The USA	(6)
	- Fair relative declines in the utilization of general laboratory tests but not radiology tests		
	- Reduction in emergency department visits for men at all severity levels (low, intermediate, and high severity visits)		
	- Reduction in low severity emergency visits for women although being changeless in intermediate and high severity visits	The USA	(15)
	- A relative decrease in hospitalizations for men in the first year, and then a relative increase in hospitalizations between the first and second year after opting for high deductible health plans.		
Impacts on utilization of the insured	- A decrease in high-severity emergency department visits by low socioeconomic status enrollees	The USA	(16)
	- Hospitalization decrease in people whose socioeconomic status were low in the first year after opting in the plan followed by an increase in the second year.		
	-NO significant difference in the health-care decision-making strategies between people with low deductible and high deductible plans	The USA	(17)
	-Changing the plan to a high deductible health plan by the insured with 3 wide-spread chronic diseases did not decrease the use of crucial medicine	The USA	(18)
	- Enrollment in high-deductible health plans caused reduction in the use of the emergency room but increase in prescription medicine use	The USA	(19)
	-Increase in utilization was more probable in the insured with chronic illnesses and people who could choose the plans		
	- The deficiency of the insured’s information about deductible plan limits the impact of deductibles on utilization	The USA	(20)
	- Using breast, cervical and colorectal cancer screening as completely covered by insurance instead of using tests subject to deductible	The USA	(21)
	- Deductible plan lowers the use of health services by German enrollees	Germany	(22)
	- No decrease in moral hazard over time without risk rating the insured in the deductible plan	The Netherland	(23)
	- The most price elasticity for general physician visits, the least for specialists care, and no effects on hospitalization after opting for the deductible plan	The Netherland	(24)
	-increase in out of pocket spending among adults with chronic diseases	The USA	(25)
	- Higher OOP burdens for low-income groups compared to those in the higher income groups	The USA	(26)
Financial impacts on the insured	- The financial profitability of voluntary deductible for almost half of the Dutch insured	The Netherland	(27)
	-The financial profitability of deductibles for men, the young and healthy insured		
	- About half of the families with chronic diseases opting for high-deductible plans		(28)
	confronted financial burden due to health care costs. This quantity was almost half for the insured of low deductible health plans	The USA	
	- No difference in the total outpatient costs	The USA	(19)
	- High deductible health plans is profitable for young and healthy people		
	- A new kind of deductible(shifted deductible) may decline OOP costs	The Netherland	(29)
Financial impacts on health insurance organization	-Saving a total of €1.71 million in benefits by Germany’s third-largest social health insurance fund (Techniker Krankenkasse) as a result of a deductible pilot scheme	Germany	(22)
	- Lower health benefit claims in the insured who opted for deductible in German health statutory insurance	Germany	(30)

**Table 3:** The most important positive and negative impacts of deductibles

***Impacts***	***Positive impacts***	***Negative impacts***
Impacts on utilizations of the insured	-decrease in utilization of different services	-higher hospitalization over time
Financial impacts on the insured persons	- being profitable for the young and healthy insured persons	-increase in out of pocket burdens specially for people with chronic diseases
	-the probability of decrease in out of pocket costs by a new kind of deductible (shifted deductible)	and low income
Financial impacts on the insurance organizations	-lower health benefit claims by the insured people and increase in financial profitability of health insurance organization	None

## Discussion

The present study tried to present a comprehensive review of the positive and negative effects of implementing deductible plans by studying the previous documents on the various dimensions of the plans.

Various researches on implementation of deductibles in the studied countries show that the most important goal of implementing these plans is to prevent moral hazard by reducing the use of unnecessary health care services (27, 29). The findings of several studies suggest that the use of health care services has decreased with the introduction of deductibles, so that decrease in referrals to specialists in two studies, (13, 22), slight reduction in medication consumption in one study (11), reduction in diagnostic imaging in one study (14), decrease in the number of visits by general practitioners in two studies (10, 22), and a modest decrease in physicians visits for chronic diseases as well as general tests in one study, Reddy (6), were reported.

On the other hand, some studies have not evaluated the use of deductibles significantly effective in reducing health care utilization. For instance, lack of a significant difference in physician visits for acute illnesses and preventive diagnostic tests, as well as being ineffective on the number of radiographic imaging (6), lack of a significant reduction in the use of essential medications for three common chronic conditions (Diabetes, blood pressure and asthma) (18), lack of change in the overall costs of outpatient services, and the likelihood of an increase in the consumption of services for chronic illnesses, as well as the medical costs of those who can choose their insurance plan (19), and lack of difference in the use of cancer screening tests (21), are reported as evidences supporting lack of expected association between deductibles and the use of above-mentioned services by insured persons.

Notably, although reduction in the use of health care services due to implementation of deductibles can potentially prevent moral hazard and consequently lead to cost savings, this can encourage people not to use essential health care services with preventive aspect, and this, in turn, may endanger people’s health in the medium and long term. In other words, it is necessary to distinguish between proper and rational reduction in the use of health care services and improper and irrational use. In this regard, in a study on men and women (15), after implementation of deductibles, the rate of the men referring to the emergency department reduced at all levels of “low risk”, “moderate risk” and “high risk” but regarding women, this rate decreased only in “low risk” level. Interestingly, although men in the first year after registering in deductible plan used less inpatient care services compared to the past, this trend was different in the second year, so that they used more hospitalization services compared to the time before registering in the plan. In addition, Agarwal (12) concluded from his study that after registering in deductible plan, individuals’ use of health preventive services and essential medicines declined.

Apart from cost reasons, one of the most important factors in this regard can be people awareness about their health status as well as the deductible plan. This issue clearly is reflected in Wharam (16) study. In this study, people from lower socioeconomic backgrounds, in the first year after registering in deductible plans, experienced a decline in hospitalization and in the second year experienced an increase in hospitalization; but such changes were not found in people from higher socioeconomic backgrounds. Similarly, the findings of Reed (20) also confirmed the fact that individuals’ knowledge about deductibles structure and services covered by this plan influences utilization of the services, so that it can limit the achievement to the goals of implementing deductibles plans. In another study, regarding adoption of health care decision-making strategies no significant difference was observed between those registered in high-cost sharing plans and those registered in low-cost sharing plans (17). According to Gupta, presence of relevant information on deductibles is highly important and necessary to decide on how to use health care services.

Although there is a close, bilateral relationship between the utilization of health care services and the financial dimensions resulting from implementing the deductibles plans, it is necessary to address the financial implications resulting from implementation of the deductibles separately.

In this study, finding some evidence about the financial impacts of the deductibles on insured persons and, on the other hand, on insurance organizations is considerable. The average out-of-pocket expenses for patients with chronic illnesses who opted for high deductible health plans and also low deductible health plans was respectively $225 and $111 more than similar patients without deductibles (25). Nevertheless, deductible is not considered a financial barrier for chronic patients to access health care services. Correspondingly, nearly half of the families with members affected with chronic illnesses used high deductible health plans suffered financial burden related to health care services (28). This rate was 21% for families using traditional and common deductible plans. Moreover, the percentage of the families registered in high deductible health plans and had spent more than 3% of their income on health services was almost twice the percentage of families registered in traditional deductible plans and had spent more than %3 of their income on health services. Although high deductible health plans limit confronting catastrophic expenditures, some families may be left without adequate insurance cover. Similarly, the frequency of out-of-pocket payment burdens for low-income insured persons highly increased with increase in deductibles levels (26). By contrast, the frequency of these burdens for the insured people from higher income groups changes much less with an increase in deductibles levels. Regardless of the level of deductibles, for those in lower-income groups, it was much more likely to face high financial burdens due to deductibles.

Of course, some studies have reported positive effects of deductibles on the insured persons. For example, an optional deductible of €500, which started after a mandatory deductible, was beneficial for 48% of Dutch insured persons (27). In addition, the researcher concluded, in general, that deductibles are beneficial for men, the young, healthy insured persons and those who had little health expenditures in the past. In this regard, another researcher (19), states in the same way that since healthy and young people, predict they won’t need medical care services, welcome high deductible health plans in comparison with traditional common plans, because it leads to financial savings for them - through receiving premium discounts. Moreover, those who predict significant health care costs for themselves welcome deductibles, since after reaching the ceiling limit of payments for high deductible health plans no longer are required to pay.

The other issue worth noting regarding financial effects of deductibles on insured persons is introduction of a new type of deductible i.e. shifted deductible (29). In this type of deductible, paying deductible does not begin from the starting point of health services costs. The researcher believes this type of deductible reduces the likelihood that the health costs of high-risk individuals exceed the upper limit of deductibles, and, on the other hand, increases the price sensitivity of these types of insured persons to their health care services and ultimately, it can reduce their out-of-pocket costs. Van Winssen et al, referred to shifted deductible as an effective strategy to increase the number of individuals opting for voluntary deductibles, and consequently, reduce moral hazard (27).

Apart from financial impacts of deductibles on the insured persons, insurance organizations are also affected by deductibles. For example, during one year, insurance claims were lower for those paid deductibles in comparison with those without deductibles (30). In addition, the results of the study revealed that the claims for health insurance decrease with increase in deductible.

The usefulness of the deductible implementation for insurance organizations was shown in another study in Germany (22). The findings of the study showed that the third German Social Insurance Fund, Techniker Krankenkasse (TK), after implementation of a pilot deductible plan, in 2003, saved €645,000 for physician visit costs among the total covered population. Moreover, the costs of the fund reduced regarding hospital treatments, a variety of preventive examinations and tests, and medications, so that, in sum, implementing this pilot plan resulted in savings 1.71 million euros for the fund.

Overall, since deductibles reduce the need for health care services and consequently reduce the necessity of insurance organizations participation in initial costs of insured persons, they lead to considerable savings for these organizations through decrease in unnecessary services, as well as reduction in administrative costs.

## Conclusion

In recent years the issues of using deductible plans and reforming the method of implementing them in health insurance systems of some developed countries have always been raised.

Some of the reforms, among the others, are implementation of high deductible health plans in the United States, and the shifted deductible plan proposed in the Netherlands. Each of these plans has its advantages and disadvantages for the insured persons and insurance organizations in terms of financial dimensions as well as utilization of health services, and explicitly none of these plans can be flawless.

Given the increasing costs of health systems and the potential moral hazard of insured persons, it seems these systems sooner or later should inevitably move towards new cost-sharing plans, including deductibles.

## Ethical considerations

Ethical issues (Including plagiarism, informed consent, misconduct, data fabrication and/or falsification, double publication and/or submission, redundancy, etc.) have been completely observed by the authors.
